# Mesh placement and patient-reported outcomes in primary ventral hernia repair: a nationwide survey- and register-based study

**DOI:** 10.1007/s00464-026-12911-5

**Published:** 2026-05-22

**Authors:** Usamah Ahmed, Hugin Reistrup, Anders Gram-Hanssen, Jacob Rosenberg, Laus Wolsing Wullum, Jason Joe Baker

**Affiliations:** 1 Center for Perioperative Optimization, Department of Surgery, Copenhagen University Hospital - Herlev and Gentofte, Borgmester Ib Juuls Vej 1, DK-2730 Herlev, Denmark; 2 Sanos Group ApS, Telefonvej 8D, DK-2860 Søborg, Denmark

**Keywords:** Primary ventral hernia, Mesh, Patient-reported outcomes, Chronic pain, Foreign body sensation

## Abstract

**Background:**

Mesh repair reduces recurrence risk, but evidence regarding patient-reported outcomes (PROs) across mesh placements remains limited. This study aimed to investigate the association between mesh placement and PROs in primary ventral hernia repair, and secondarily the prevalence of severe chronic pain and foreign body sensation.

**Methods:**

This nationwide survey- and register-based study, part of the AFTERHERNIA project in Denmark, linked electronic survey responses with operative data from the Danish Ventral Hernia Database. Adults (≥ 18 years) who underwent elective primary (umbilical or epigastric) ventral hernia repair with mesh between January 1, 2014, and March 31, 2024, were included, limited to defects < 10 cm. PROs were assessed using the validated, ventral hernia-specific Abdominal Hernia-Q (AHQ) questionnaire.

**Results:**

Out of 13,327 eligible patients, 10,869 (82%) responded (median age, 54 years; 71% male). Compared with onlay, preperitoneal repair had a better AHQ sum score (58.7 vs 57.7; mean difference, 0.9; 95% CI, 0.6 to 1.2; *P* < .001), lower prevalence of severe chronic pain (8.7% vs 11.3%; difference, −2.6%; 95% CI, −4.0% to −1.2%; *P* < .001), and lower prevalence of foreign body sensation (20.1% vs 23.9%; difference, −3.8%; 95% CI, −5.7% to −1.8%; *P* < .001). Findings were consistent across subgroups and sensitivity analyses.

**Conclusions:**

PROs were generally good across mesh placements. Preperitoneal mesh placement was associated with slightly better PROs compared with other placements, although the difference was small and unlikely to be clinically meaningful. However, it was associated with a lower prevalence of severe chronic pain and foreign body sensation compared with onlay and intraperitoneal mesh.

**Graphical abstract:**

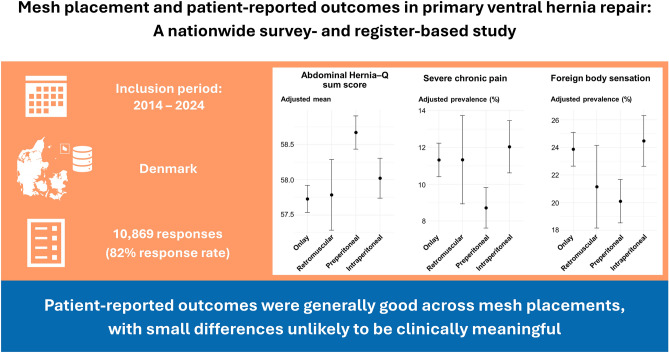

**Supplementary Information:**

The online version contains supplementary material available at 10.1007/s00464-026-12911-5.

Mesh repair is recommended for primary ventral hernia repair, as it provides structural reinforcement of the abdominal wall and lowers the risk of recurrence compared with suture-only repair [1]. Mesh can be placed in different anatomical layers of the abdominal wall [2], with the most commonly used placements being onlay, retromuscular, preperitoneal, and intraperitoneal [3]. The choice of mesh placement may not be trivial, as different anatomical layers have been associated with potentially different outcomes [4]. Surgical outcomes such as recurrence and surgical site infections have been examined in relation to mesh placement, with some evidence suggesting advantages for preperitoneal or retromuscular placement [5]. While these clinical outcomes are important indicators of surgical success, they do not fully capture the patient experience [6]. Patient-reported outcomes (PROs), including pain, foreign body sensation, function, and quality of life, are increasingly recognized as important measures, providing essential insight into the long-term success of hernia repair from the patient’s perspective [7, 8].

Despite this, evidence on PROs in relation to mesh placement remains limited. A recent systematic review highlighted the very limited evidence available, particularly concerning the risk of chronic pain and foreign body sensation across different mesh placements [9]. Nevertheless, a recent international survey showed that many surgeons favor preperitoneal and retromuscular mesh placement [10], a preference supported by current guidelines [11], but whether these placements translate into superior PROs remains unknown.

This study aimed to investigate the association between mesh placement and PROs in adults undergoing elective primary ventral hernia repair, with a secondary focus on the prevalence of severe chronic pain and foreign body sensation.

## Methods

### Study design

This nationwide Danish survey- and register-based study used prospectively collected data from the Danish Ventral Hernia Database [12], linked with PROs from the Abdominal Hernia-Q (AHQ) questionnaire [13] from the AFTERHERNIA project [14]. The study was reported in accordance with the Reporting of studies Conducted using Observational Routinely-collected health Data (RECORD) and Consensus-Based Checklist for Reporting of Survey Studies (CROSS) guidelines [15, 16]. A statistical analysis plan was prepared before analyses and uploaded to an open repository [17].

### Setting

All patients who underwent ventral hernia repair in Denmark between January 1, 2014, and March 31, 2024, were identified through the Danish National Patient Register [18]. They were invited to complete an electronic survey based on the AHQ via Digital Post, a secure national platform for communication between citizens and public authorities in Denmark [19], used by 95% of individuals aged 15 years or older [20]. Data were extracted from the AFTERHERNIA cohort on August 15, 2025, and all survey responses completed up to that date were included in the analyses. Further details on survey design, registry data, and linkage methodology are available in the published AFTERHERNIA protocol [14].

### Registers

In Denmark, all residents are assigned a unique personal identification number, which is used for all contacts within the healthcare system and enables linkage across national registers [21]. The Danish National Patient Register provides a nationwide longitudinal record of all hospital and clinic contacts in both the public and private sectors [18]. These data were linked to the Danish Civil Registration System, which holds continuously updated information on name, address, and changes such as death and emigration [21]. Further linkage was made with the Danish Ventral Hernia Database [12], which had a registration rate of 89% in 2023 [22], and has been validated against patient records with 89–99% accuracy [23]. Data are entered directly by operating surgeons from both public and private hospitals, including clinics, and registration is mandatory [12]. The Danish Ventral Hernia Database records patient and hernia characteristics, along with operative details such as surgical approach, mesh use, and mesh placement [24]. Finally, the Danish Authorization Register was used to obtain information on surgeons, including surgeon ID, specialty, and date of specialization [25].

### Survey

The AFTERHERNIA project used the AHQ, a patient-reported outcome measure (PROM) validated for ventral hernia surgery [13] and available in Danish [26]. In this project, only the Danish postoperative questionnaire was administered. If more than one repair had been performed, responses referred to the most recent procedure. All patients received the postoperative AHQ, which covers seven domains: expectations, self and others, surgeon and surgical team, sensation, function, appearance, and overall satisfaction [13]. The questionnaire comprises 16 items scored on a 4-point Likert scale, with sum scores ranging from 16 to 64 (higher scores indicating better outcomes). The AHQ was supplemented with additional items on suspicion of recurrence, height, weight, and lifestyle factors. The survey was administered through the secure Research Electronic Data Capture (REDCap) platform [27] as a closed survey, with each participant receiving a unique, personal link. Initial invitations were distributed in batches between August and December 2024, followed by up to three electronic reminders at one-week intervals. Non-responders after the third reminder were contacted by telephone.

### Participants

Inclusion criteria were adults (≥ 18 years old) who underwent elective primary ventral hernia repair (umbilical or epigastric) in Denmark between January 1, 2014, and March 31, 2024, with a hernia defect width < 10 cm and mesh placement in onlay, retromuscular, preperitoneal, or intraperitoneal position [28]. Exclusion criteria were divided into register- and survey-based exclusions. Register-based exclusions included procedures not registered in the Danish Ventral Hernia Database (due to missing information on mesh placement), first repair registered as a recurrence repair, emergency repairs, other hernia types, other mesh placements, and cases involving component separation. Survey-based exclusions included deceased or emigrated patients, patients exempt from Digital Post [19], insufficient Danish language skills, cognitive deficits (e.g., dementia), physical handicaps (e.g., blindness, paralysis), patients feeling unable to answer due to other medical conditions making it impossible to distinguish symptoms (such as late stage cancer or a following laparotomy in the same area as the repair), non-disclosure of address, and follow-up shorter than six months. As part of the screening process, patients who denied having undergone ventral hernia surgery were also excluded. Patients were followed from the date of operation until the date of survey response.

### Data sources and measurements

The primary outcome was the AHQ sum score, and secondary outcomes were the prevalence of severe chronic pain (AHQ item 1a) and foreign body sensation (AHQ item 1b). Severe chronic pain and foreign body sensation were analyzed as binary variables, where a response of 4 indicated “no” and responses of 1 to 3 indicated “yes.”

Perioperative details were extracted from the Danish Ventral Hernia Database, including patient and hernia characteristics, operative details, and surgeon ID. Hernia defect width was categorized according to European Hernia Society and American Hernia Society guidelines as small (≤ 1 cm), medium (> 1 to ≤ 4 cm), or large (> 4 to < 10 cm) [11]. Comorbidity burden was assessed using the Charlson Comorbidity Index [29], and patients scoring ≥ 4 were grouped into a single category. Information on surgeon ID and supervision of operations was introduced into the Danish Ventral Hernia Database in 2017, resulting in missing data for earlier years. Surgeons were classified as specialists if they were registered in general or plastic surgery and had completed specialization at the time of repair. Implausible height or weight values were considered invalid and treated as missing data.

### Statistics

Categorical data were presented as counts and percentages. Continuous data were assessed for normality using visual inspection of histograms and quantile–quantile (Q–Q) plots. As most continuous data were not normally distributed, they were reported as medians with interquartile ranges. The primary analysis estimated marginal mean AHQ sum scores for each mesh placement using adjusted linear regression with standardization (g-computation) to control for confounding. Marginal standardization provides adjusted marginal average values (e.g., means or prevalence) for each placement [30]. Absolute differences in marginal means were calculated using onlay placement as the reference. Models were adjusted for age, sex [31, 32], hernia defect width (continuous) [33], Charlson Comorbidity Index [29], body mass index [34], follow-up time [35], and smoking status [36]. Secondary analyses assessed the prevalence of severe chronic pain and foreign body sensation using adjusted logistic regression with the same covariates, standardized to estimate marginal prevalence and prevalence differences. Model assumptions were assessed by visual inspection of residual plots. Robust variance estimation was applied to ensure valid confidence intervals and P values in case of model misspecification. Analyses were based on complete cases without imputation. Statistical significance was defined as two-sided *P* < 0.05. All data cleaning and analyses were conducted in R, version 4.4.2 [37], using the Targeted package, version 0.5 [38], and performed by professional statisticians.

Subgroup and sensitivity analyses were performed for the AHQ sum score. Subgroup analyses were conducted for small and medium hernia defect widths and for open and laparoscopic approaches. Analyses for large defects and robotic repairs were not feasible because of limited sample size and covariate coverage. Sensitivity analyses included: removal of AHQ domains unrelated to mesh placement (Surgical Team and Preparedness: items 3a, 4a, 5a, 6a, and 7a), exclusion of patients with self-reported recurrence, exclusion of patients treated with Physiomesh™, which has been withdrawn from the market due to high recurrence rates [39], stratification by surgeon specialization (specialist vs non-specialist vs supervised non-specialist), restriction to IPOM+ repairs (intraperitoneal mesh with defect closure) [40], and inverse probability weighting targeting the same estimand using propensity scores fitted with the same covariates as the primary analysis to assess robustness to model misspecification [30, 41].

### Ethical considerations and approvals

All participants provided informed consent at the time of survey participation. The study was approved by the Danish Healthcare Quality Institute, the Danish Data Protection Agency (journal no. p−2023−14805), the Capital Region of Denmark’s Committees on Health Research Ethics (R−24033183), and was registered with the Danish Health Data Authority (FSEID−00006834).

## Results

### Selection of participants

**Fig. 1:**
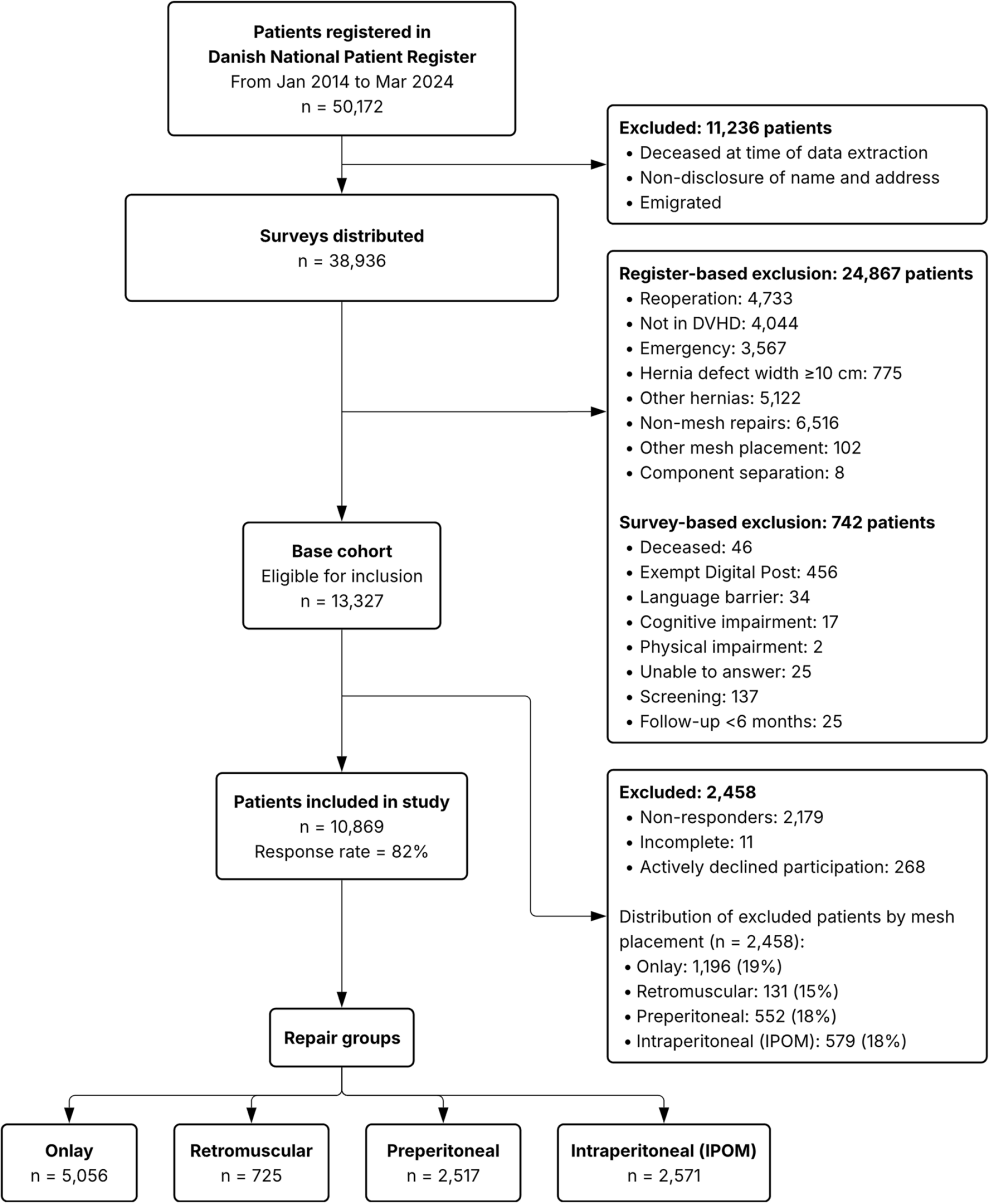
Flowchart of patient selection. The base cohort was derived from the AFTERHERNIA project and refined through eligibility criteria. The base cohort included only patients relevant to the study objectives, and the response rate was calculated from all eligible patients. *DVHD*, Danish Ventral Hernia Database; *IPOM*, intraperitoneal onlay mesh; *n*, number

In the AFTERHERNIA project, 38,936 patients were invited to participate (Fig. 1). After applying study-specific eligibility criteria, 13,327 patients formed the base cohort relevant to the project, of whom 10,869 completed the survey, yielding a response rate of 82%. The distribution of non-responders was similar across mesh placement groups (15 to 19%).

### Characteristics

Most patients had mesh placed onlay (47%), whereas retromuscular placement was least common (7%) (Table 1). Age, sex, Charlson Comorbidity Index, and smoking status were similarly distributed across groups. The majority of hernias repaired were medium-sized (> 1 to ≤ 4 cm). For small (≤ 1 cm) and medium (> 1 to ≤ 4 cm) defects, onlay placement was most common, followed by preperitoneal and intraperitoneal mesh. Retromuscular and intraperitoneal meshes were more frequently used for large defects (> 4 to < 10 cm) compared with preperitoneal and onlay. Intraperitoneal mesh predominated in laparoscopic repairs, retromuscular was evenly distributed between open and robotic approaches, and two-thirds of preperitoneal repairs were performed with open approach. Onlay was exclusively performed via open approach. At least nine out of ten repairs were performed by specialists or supervised surgeons, except for onlay where this proportion was approximately eight out of ten. Median follow-up was three to four years longer for intraperitoneal compared with other mesh placements. Patients in the intraperitoneal group also reported a 4–5% higher suspicion of recurrence.

**Table 1:** Baseline characteristics of patients, perioperative details, and crude outcomes

	Onlay (*n* = 5,056)	Retromuscular (*n* = 725)	Preperitoneal (*n* = 2,517)	Intraperitoneal (*n* = 2,571)
Age (years), median [IQR]	54 [44–63]	56 [48–65]	54 [44–62]	54 [46–63]
Female	1,418 (28)	232 (32)	676 (27)	814 (32)
Charlson comorbidity index				
0	3,750 (74)	511 (70)	1,892 (75)	1,840 (72)
1	668 (13)	112 (15)	350 (14)	427 (17)
2	397 (8)	67 (9)	176 (7)	189 (7)
3	129 (3)	25 (3)	52 (2)	62 (2)
≥ 4	112 (2)	10 (1)	47 (2)	53 (2)
Smoking status				
Active	753 (15)	95 (13)	332 (13)	387 (15)
Previous	1,245 (25)	175 (24)	707 (28)	774 (30)
Body mass index				
< 18.5	24 (1)	4 (1)	11 (< 1)	10 (< 1)
18.5–24.9	1,183 (24)	79 (11)	569 (23)	394 (16)
25–29.9	2,214 (45)	271 (38)	1,091 (44)	945 (38)
30–34.9	1,178 (24)	217 (30)	607 (24)	740 (29)
35–39.9	279 (6)	96 (13)	157 (6)	306 (12)
≥ 40	67 (1)	47 (7)	47 (2)	122 (5)
Missing	111 (2)	11 (2)	35 (1)	54 (2)
Hernia defect width, cm				
≤ 1	2,419 (48)	84 (12)	788 (31)	478 (19)
> 1 to ≤ 4	2,581 (51)	510 (70)	1,706 (68)	1,964 (76)
> 4 to < 10	56 (1)	131 (18)	23 (1)	129 (5)
Specialist surgeon				
Yes	865 (30)	200 (62)	324 (47)	505 (64)
No	516 (18)	31 (10)	52 (8)	53 (7)
No, but supervised	1,549 (53)	92 (28)	312 (45)	236 (30)
Missing	2,126 (42)	402 (55)	1,829 (73)	1,777 (69)
Surgical approach				
Open	5,056 (100)	344 (47)	1,577 (63)	754 (29)
Laparoscopic	0 (0)	0 (0)	889 (35)	1,813 (71)
Robot-assisted	0 (0)	381 (53)	51 (2.0)	4 (< 1)
Fixation methods				
Sutures	4,902 (97)	248 (34)	1,653 (66)	757 (29)
Tackers	7 (< 1)	1 (< 1)	683 (27)	1,345 (52)
Sutures and tackers	0 (0)	0 (0)	6 (< 1)	408 (16)
Self-fixating	120 (2)	267 (37)	117 (5)	0 (0)
Other	27 (1)	209 (29)	58 (2)	61 (2)
Outcomes				
Follow-up (years), median [IQR]	3.8 [1.9–6.0]	3.5 [1.9–5.8]	4.6 [2.2–7.2]	7.7 [5.8–9.2]
AHQ sum score, median [IQR]	60 [55–63]	60 [55–63]	61 [56–63]	60 [55–63]
Severe chronic pain	599 (12)	86 (12)	221 (9)	288 (11)
Foreign body sensation	1,267 (25)	157 (22)	518 (21)	561 (22)
Self-reported recurrence	535 (11)	82 (11)	249 (10)	381 (15)

Values are n (%) unless otherwise indicated. For fixation methods, “other” includes none, glue, and mixed techniques. *AHQ* Abdominal Hernia-Q, *IQR* interquartile range, *n* number

### AHQ sum score

**Fig. 2:**
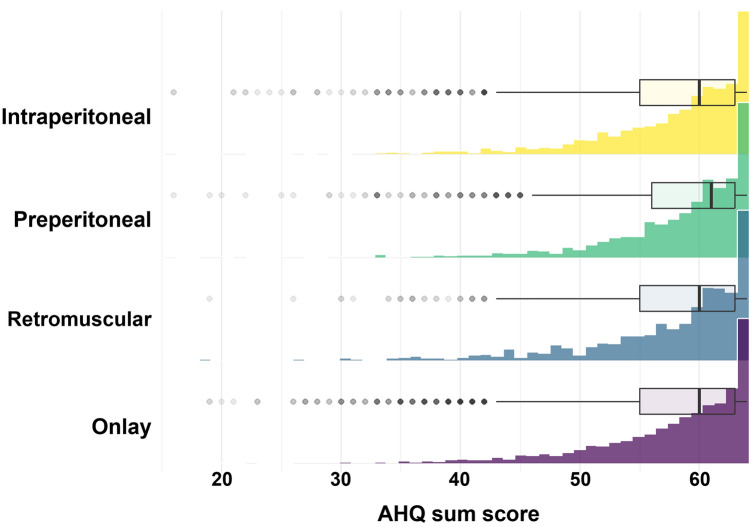
Abdominal Hernia-Q (AHQ) sum score stratified by mesh placement. Column heights indicate the number of patients at each AHQ sum score. Overlaid box plots show median AHQ sum score and interquartile range (25th–75th percentiles), with outliers as dots. The AHQ sum score ranges from 16 to 64, with higher scores indicating better outcomes. Most patients reported high scores across all mesh placements

**Fig. 3:**
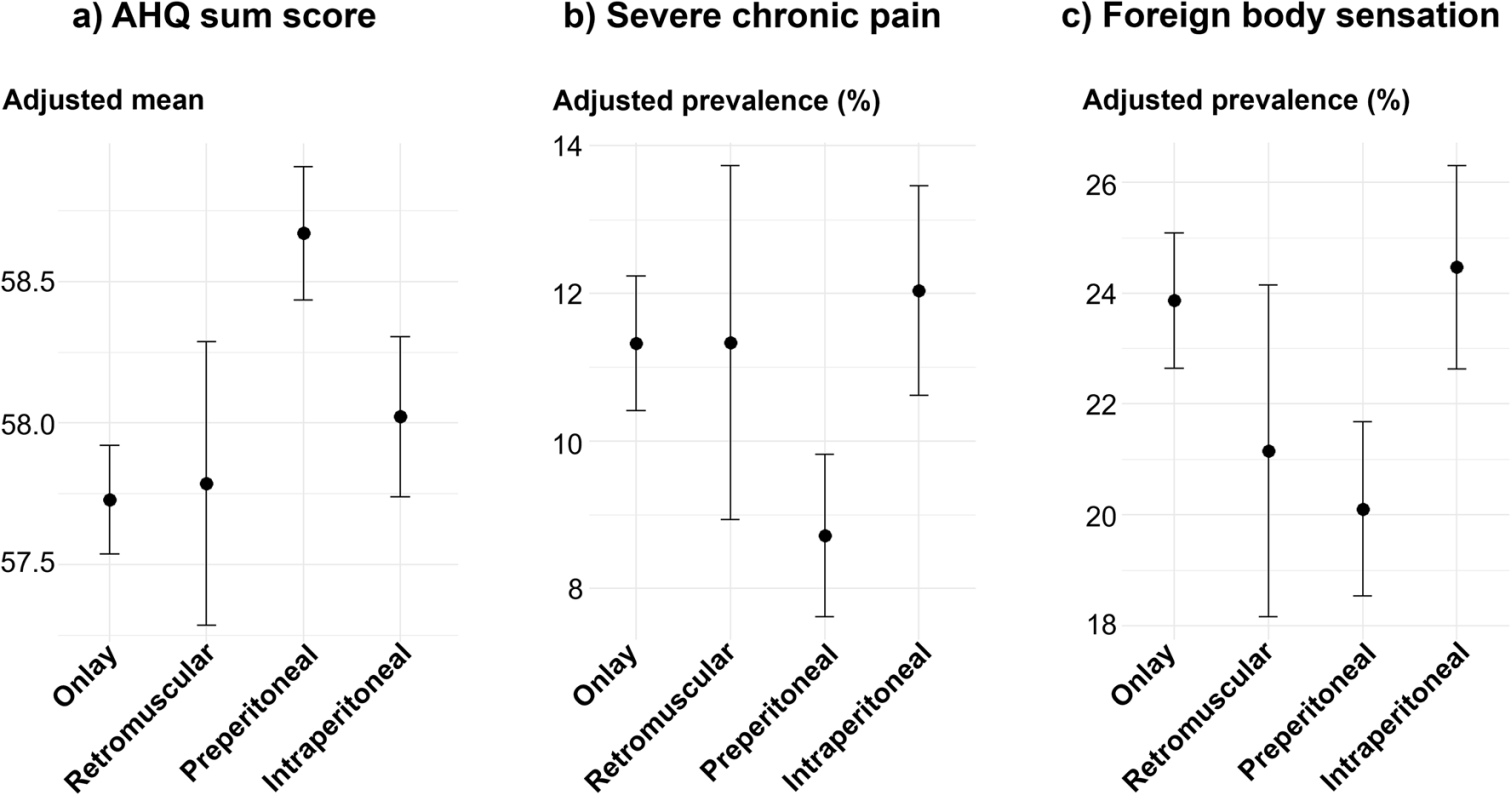
Adjusted analyses of patient-reported outcomes by mesh placement. Dot plots show (**a**) Abdominal Hernia-Q (AHQ) sum scores, (**b**) prevalence of severe chronic pain, and (**c**) prevalence of foreign body sensation. Linear regression was used for the AHQ sum scores, and logistic regression for the prevalence of severe chronic pain and foreign body sensation, followed by standardization. Dots represent adjusted means or prevalence, and error bars indicate 95% confidence intervals

The unadjusted AHQ sum scores were high and similar across all mesh placements (Table 1, Fig. 2). The lower 25th percentile indicated that 25% of patients had an AHQ sum score of ≤ 56 in the preperitoneal group and ≤ 55 in the other placements. In adjusted analyses, only preperitoneal mesh placement was associated with higher AHQ sum scores compared with onlay placement (58.7 vs 57.7; mean difference, 0.9; 95% CI, 0.6 to 1.2; *P* < 0.001) (Table 2, Fig. 3a).

**Table 2:** Adjusted mean/prevalence and difference by mesh placement

	Adjusted mean/prevalence	Adjusted difference	
	Estimate (95% CI)	Estimate (95% CI)	*p*-value
AHQ sum score			
Onlay	57.7 (57.5 to 57.9)	Reference	–
Retromuscular	57.8 (57.3 to 58.3)	0.1 (−0.5 to 0.6)	.832
Preperitoneal	58.7 (58.4 to 58.9)	0.9 (0.6 to 1.2)	< .001
Intraperitoneal	58.0 (57.7 to 58.3)	0.3 (−0.1 to 0.6)	.103
Severe chronic pain (%)			
Onlay	11.3 (10.4 to 12.2)	Reference	–
Retromuscular	11.3 (8.9 to 13.7)	0.0 (−2.6 to 2.6)	.996
Preperitoneal	8.7 (7.6 to 9.8)	−2.6 (−4.0 to −1.2)	< .001
Intraperitoneal	12.0 (10.6 to 13.5)	0.7 (−1.1 to 2.5)	.428
Foreign body sensation (%)			
Onlay	23.9 (22.6 to 25.1)	Reference	–
Retromuscular	21.2 (18.2 to 24.2)	−2.7 (−5.9 to 0.5)	.100
Preperitoneal	20.1 (18.5 to 21.7)	−3.8 (−5.7 to −1.8)	< .001
Intraperitoneal	24.5 (22.6 to 26.3)	0.6 (−1.7 to 2.9)	.607

Linear regression was applied for the AHQ sum score, and logistic regression for the prevalence of severe chronic pain and foreign body sensation. Models were adjusted for age, sex, hernia defect width, Charlson Comorbidity Index, body mass index (BMI), smoking status, and follow-up. Adjusted means/prevalences and differences are reported; unadjusted values are available in Table S1. *AHQ* Abdominal Hernia-Q, *CI* confidence interval

**Fig. 4:**
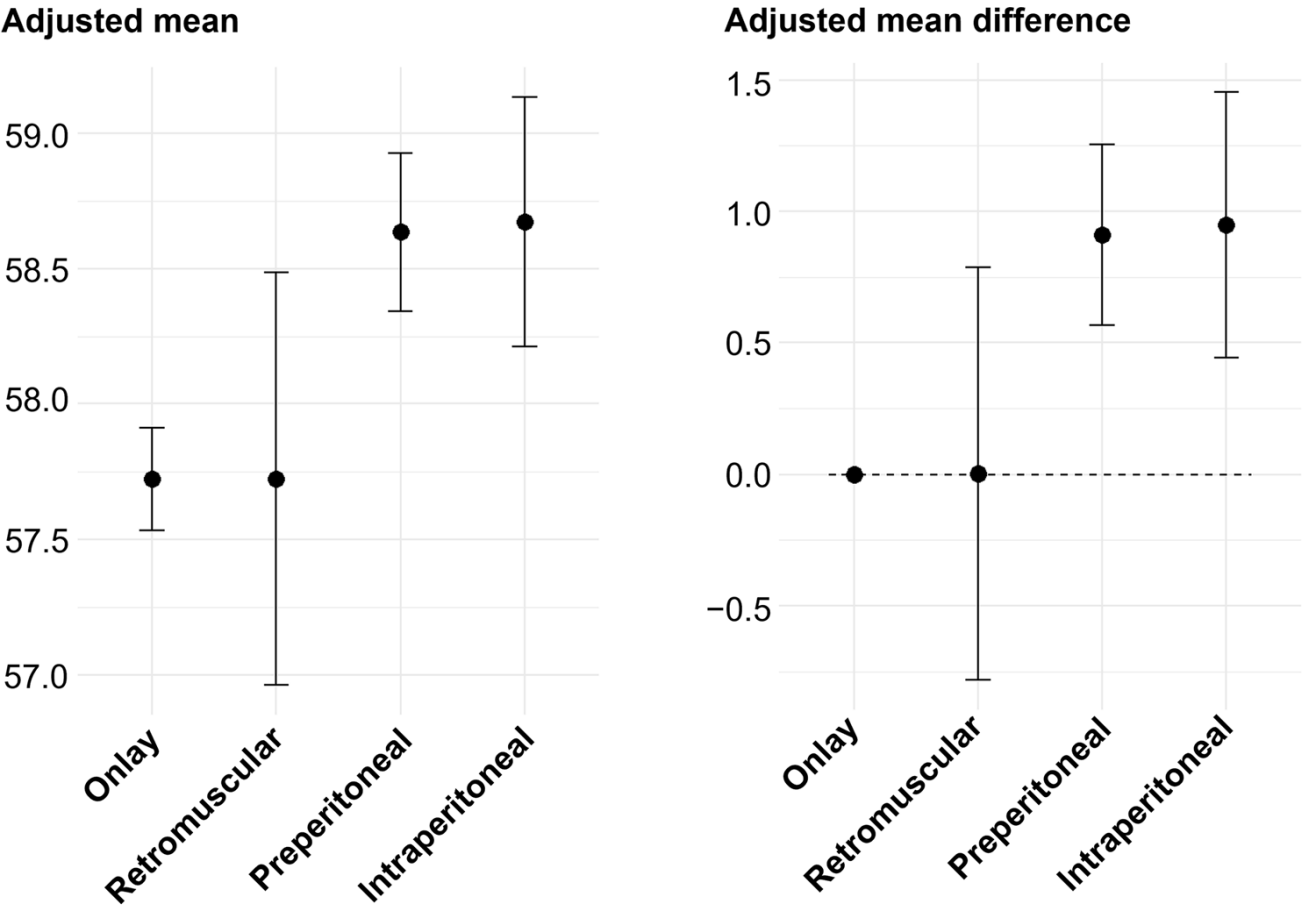
Adjusted analyses of patient-reported outcomes by mesh placement in open repairs. Dots indicate adjusted means (left) and adjusted mean differences (right) in Abdominal Hernia-Q sum scores. Error bars represent 95% confidence intervals

Subgroup analyses by surgical approach (Table 3, Fig. 4) and hernia defect width (Table 4) found results similar to the main analysis, with minimal differences in adjusted mean AHQ sum scores. Sensitivity analyses likewise supported the main findings, with preperitoneal placement consistently scoring slightly higher than onlay (Tables S2–S7). Intraperitoneal placement also showed slightly higher AHQ sum scores than onlay when AHQ items related to Surgical Team and Preparedness were excluded from the sum score (Table S2), and when patients with self-reported recurrence were excluded (Table S3). Results were confirmed by inverse probability weighting analyses, which supported the robustness of the main findings (Table S7).

**Table 3:** Abdominal Hernia-Q (AHQ) sum scores by mesh placement, stratified according to surgical approach

	Adjusted mean	Adjusted difference	
	Estimate (95% CI)	Estimate (95% CI)	*p*-value
Open			
Onlay	57.7 (57.5 to 57.9)	Reference	–
Retromuscular	57.7 (57.0 to 58.5)	0.0 (−0.8 to 0.8)	.995
Preperitoneal	58.6 (58.3 to 58.9)	0.9 (0.6 to 1.3)	< .001
Intraperitoneal	58.7 (58.2 to 59.1)	0.9 (0.4 to 1.5)	< .001
Laparoscopic			
Preperitoneal	58.7 (58.2 to 59.1)	Reference	–
Intraperitoneal	57.8 (57.4 to 58.1)	−0.9 (−1.5 to −0.3)	.002

In the open surgery subgroup, both preperitoneal and intraperitoneal placements were associated with higher AHQ sum scores compared with onlay. In the laparoscopic subgroup, preperitoneal placement was associated with higher AHQ sum scores than intraperitoneal. Laparoscopic subgroup analysis was not feasible for all mesh placements because no laparoscopically performed onlay and retromuscular repairs were available. Robot-assisted subgroup analysis was not feasible owing to very few cases across all mesh placements except retromuscular, which did not allow full covariate adjustment. *AHQ* Abdominal Hernia-Q, *CI* confidence interval

**Table 4:** Abdominal Hernia-Q (AHQ) sum scores by mesh placement, stratified according to hernia defect width categories

	Adjusted mean	Adjusted difference	
	Estimate (95% CI)	Estimate (95% CI)	*p*-value
≤ 1 cm			
Onlay	57.2 (56.9 to 57.5)	Reference	–
Retromuscular	57.9 (56.4 to 59.3)	0.7 (−0.8 to 2.1)	.367
Preperitoneal	58.4 (58.0 to 58.9)	1.2 (0.7 to 1.7)	< .001
Intraperitoneal	57.8 (57.2 to 58.4)	0.6 (−0.1 to 1.3)	.103
> 1 to ≤ 4 cm			
Onlay	58.1 (57.8 to 58.3)	Reference	–
Retromuscular	58.1 (57.5 to 58.6)	0.0 (−0.6 to 0.6)	.999
Preperitoneal	58.9 (58.6 to 59.2)	0.8 (0.4 to 1.2)	< .001
Intraperitoneal	58.3 (57.9 to 58.6)	0.2 (−0.3 to 0.6)	.435

Findings were consistent with the main analysis. Modeling of large hernias (> 4 to < 10 cm) was not feasible owing to limited sample size with the specified covariates. *AHQ* Abdominal Hernia-Q, *CI* confidence interval

### Severe chronic pain

Across all mesh placements, the adjusted prevalence ranged from 8.7% to 12.0%. Adjusted analyses showed that only preperitoneal mesh placement was associated with a lower prevalence of severe chronic pain compared with onlay placement (8.7% vs 11.3%; difference, −2.6%; 95% CI, −4.0% to −1.2%; *P* < 0.001) (Table 2). The adjusted prevalence of severe chronic pain for preperitoneal placement was also lower than intraperitoneal (Fig. 3b).

### Foreign body sensation

Across all mesh placements, the adjusted prevalence ranged from 20.1% to 24.5%. Only preperitoneal mesh placement was associated with a lower prevalence of foreign body sensation compared with onlay (20.1% vs 23.9%; difference, −3.8%; 95% CI, −5.7% to −1.8%; *P* < 0.001) (Table 2). The adjusted prevalence of foreign body sensation for preperitoneal placement was also lower than intraperitoneal but not retromuscular (Fig. 3c).

## Discussion

In this nationwide survey- and register-based study on long-term follow-up after primary ventral hernia repair with mesh, PROs measured by the AHQ were generally good across all mesh placements. Preperitoneal placement was associated with better AHQ sum scores than other mesh placements, a pattern that persisted in subgroup and sensitivity analyses. However, the absolute differences were small and unlikely to be clinically meaningful. Preperitoneal placement was associated with approximately 3% lower prevalence of severe chronic pain and 4% lower prevalence of foreign body sensation compared with onlay and intraperitoneal placements.

Our findings add to a body of evidence that has shown that mesh placement may not have a large effect on overall PROs. A randomized clinical trial (RCT) of laparoscopic retromuscular versus intraperitoneal mesh repair found less short-term pain with retromuscular placement, but no difference by three months and similar short-term quality of life [42]. Similarly, an RCT of robotic approach comparing retromuscular with intraperitoneal mesh found no difference in pain at either 30 days or 1 year, while PRO scores were slightly better after intraperitoneal repair, although the difference was small [43, 44]. In line with these findings, an observational study with long-term follow-up (five years) compared robotic retromuscular with open preperitoneal repairs and found no differences in quality of life [45].

Regarding chronic pain, some observational cohort studies have suggested potential benefits of certain mesh placements, with less chronic pain after retromuscular than onlay repair [46] and after preperitoneal compared with intraperitoneal repair [47], though chronic pain definitions were unclear, and analyses were unadjusted. For foreign body sensation, evidence was limited to one study, which found no difference between preperitoneal and intraperitoneal placement [48], while another reported more discomfort after onlay than intraperitoneal repair [49]. By focusing specifically on severe chronic pain rather than any level of pain, our study provides stronger evidence that preperitoneal placement may modestly reduce the burden of both severe chronic pain and foreign body sensation, although the absolute differences were modest. Moreover, this study supports the widely held surgical belief that preperitoneal placement may offer favorable outcomes with respect to chronic pain and foreign body sensation [10]. By demonstrating measurable, though modest, advantages, it provides empirical evidence for what has often been based largely on surgical intuition and experience.

### Strengths and limitations

This study is strengthened by focusing on PROs, including severe chronic pain and foreign body sensation, providing population-level estimates that clinicians can use for surgical decision-making. The study included all eligible patients in Denmark over a ten-year period, leveraging the comprehensive coverage of the Danish Ventral Hernia Database with mandatory registration and high data accuracy, which minimizes selection bias and ensures high external validity. The nationwide electronic delivery through Digital Post [19] combined with a structured follow-up strategy, enhanced participation, and yielded a high response rate of 82% while minimizing non-response. The balanced distribution of non-responders across mesh placements further suggests that non-response was unlikely to bias mesh comparisons. The robustness of the findings is supported by consistent results across multiple sensitivity analyses, indicating that observed associations were not dependent on specific modeling assumptions or analytic choices. However, interpretation of differences in mean AHQ sum scores by mesh placement is limited by the absence of an established minimal clinically important difference (MCID). Consequently, it cannot be determined with certainty whether the observed difference is clinically relevant, although a mean difference of 0.9 is very unlikely to be clinically meaningful. Moreover, the AHQ item on foreign body sensation most likely captures a general internal sensation rather than a mesh-specific sensation. Furthermore, it is possible that some AHQ items may be vulnerable to recall bias or misinterpretation, and self-reported recurrence may be prone to misclassification compared with clinical verification [50]. Although survey-based exclusions were limited and the response rate was high, selection bias cannot be excluded. Moreover, the longer follow-up in the intraperitoneal group may have led to lower reported levels of chronic pain, as pain tends to decrease over time after hernia repair [51]. Although follow-up time was adjusted for in the models, residual time-related bias may remain. The observational design means that although multiple variables were adjusted for, residual confounding from unmeasured factors or variables not included in the analyses (e.g., fixation methods [52] or mesh type [53]) may remain. However, the fixation method is closely related to the surgical approach and is therefore, at least in part, accounted for in the subgroup analysis stratified by surgical approach. In addition, preoperative (baseline) pain was not captured. Since preoperative pain is a known predictor of postoperative chronic pain [32], it is not possible to distinguish whether reported postoperative pain was new, improved, or persistent. Therefore, differences in chronic pain after surgery cannot be fully attributed to mesh placement. Consequently, the study estimates associations and should not be interpreted as demonstrating causal effects.

### Perspectives

Overall, AHQ sum scores indicate that all mesh placements are clinically acceptable, but when focusing on severe chronic pain and foreign body sensation, preperitoneal placement may be preferred. However, the observed differences were modest and could reflect variation between surgeons. With respect to PROs, mesh placement should therefore be guided primarily by the technique with which the surgeon is most proficient. However, when feasible, preperitoneal placement may be preferred when this technique is within the surgeon’s expertise. Other technical aspects of ventral hernia repair, including fixation methods, suturing, and surgical approach, should also be investigated for their effect on PROs. Given the relatively high prevalence of severe chronic pain and foreign body sensation after primary ventral hernia repair, future studies should clarify how technical factors beyond mesh placement influence PROs.

## Conclusion

PROs measured by AHQ were generally good across mesh placements. Preperitoneal mesh placement was associated with slightly better AHQ sum scores than other mesh placements, although the difference was small and unlikely to be clinically meaningful. However, preperitoneal placement was associated with a lower prevalence of severe chronic pain and foreign body sensation compared with onlay and intraperitoneal mesh placement.

## Supplementary Information

Below is the link to the electronic supplementary material.

Supplementary file1 (PDF 358 KB)

## Data Availability

Due to Danish legislation, supporting data are not available.
