# Supplementary material for: Mesh placement and patient-reported outcomes in primary ventral hernia repair: a nationwide survey- and register-based study
Source: Surg Endosc. 2026 May 22;40(7):6116–27. doi: 10.1007/s00464-026-12911-5 (PMC13369696; doi:10.1007/s00464-026-12911-5)
Supplement: Supplementary file 1 — Supplementary file1 (PDF 358 KB) [file 464_2026_12911_MOESM1_ESM.pdf]

Mesh placement and patient-reported outcomes in primary ventral hernia repair: a nationwide survey- and register-based study

Usamah Ahmed, MD, PhD[1], Hugin Reistrup, MD, PhD[1], Anders Gram-Hanssen, MD, PhD[1], Jacob Rosenberg, MD, DMSc[1], Laus Wolsing Wullum, MSc[2], Jason Joe Baker, MD, PhD[1]

[1] Center for Perioperative Optimization, Department of Surgery, Copenhagen University Hospital - Herlev and Gentofte, Borgmester Ib Juuls Vej 1, DK-2730 Herlev, Denmark
[2] Sanos Group ApS, Telefonvej 8D, DK-2860 Søborg, Denmark

Corresponding author:
Usamah Ahmed, MD, PhD
Center for Perioperative Optimization, Department of Surgery, Copenhagen University Hospital - Herlev and Gentofte, Borgmester Ib Juuls Vej 1, DK-2730 Herlev, Denmark

Supplementary Tables – Index

Table S1	*page 2*
Table S2	*page 2*
Table S3	*page 3*
Table S4	*page 3*
Table S5	*page 3*
Table S6	*page 4*
Table S7	*page 4*

Table S1

Unadjusted mean/prevalence and differences by mesh placement

	Unadjusted mean/prevalence	Unadjusted difference	
	Estimate (95% CI)	Estimate (95% CI)	p-value
**AHQ sum score**			
Onlay	57.7 (57.6 to 57.9)	Reference	–
Retromuscular	57.8 (57.3 to 58.3)	0.1 (−0.5 to 0.6)	.757
Preperitoneal	58.7 (58.4 to 58.9)	0.9 (0.6 to 1.3)	< .001
Intraperitoneal	58.0 (57.7 to 58.2)	0.2 (−0.1 to 0.5)	.208
**Severe chronic pain (%)**			
Onlay	11.6 (10.7 to 12.5)	Reference	–
Retromuscular	11.9 (9.5 to 14.3)	0.3 (−2.3 to 2.8)	.831
Preperitoneal	8.7 (7.6 to 9.8)	−3.0 (−4.4 to −1.5)	< .001
Intraperitoneal	11.2 (10.0 to 12.4)	−0.4 (−1.9 to 1.1)	.585
**Foreign body sensation (%)**			
Onlay	25.0 (23.7 to 26.2)	Reference	–
Retromuscular	21.7 (18.7 to 24.7)	−3.2 (−6.5 to 0.0)	.051
Preperitoneal	20.4 (18.8 to 22.0)	−4.5 (−6.5 to −2.5)	< .001
Intraperitoneal	21.8 (20.2 to 23.4)	−3.1 (−5.2 to −1.1)	.002

Linear regression was applied for the AHQ sum score, and logistic regression for the prevalence of severe chronic pain and foreign body sensation. AHQ, Abdominal Hernia-Q; CI, confidence interval.

Table S2

Abdominal Hernia-Q (AHQ) sum scores by mesh placement after exclusion of AHQ-domains unrelated to mesh placement (Surgical Team and Preparedness; items 3a, 4a, 5a, 6a, and 7a)

	Adjusted mean	Adjusted difference	
	Estimate (95% CI)	Estimate (95% CI)	p-value
Onlay	41.3 (41.2 to 41.4)	Reference	–
Retromuscular	41.3 (41.0 to 41.6)	0.0 (−0.3 to 0.3)	.995
Preperitoneal	42.0 (41.9 to 42.2)	0.8 (0.6 to 0.9)	< .001
Intraperitoneal	41.5 (41.3 to 41.7)	0.2 (0.1 to 0.4)	.048

The modified AHQ sum score ranges from 11 to 44, with higher scores indicating better outcomes. Findings were consistent with the main analysis; however, intraperitoneal mesh was also associated with a higher AHQ sum score compared with onlay mesh, although the difference was very small. AHQ, Abdominal Hernia-Q; CI, confidence interval.

Table S3

Abdominal Hernia-Q (AHQ) sum scores by mesh placement after exclusion of patients with self-reported recurrence

	Adjusted mean	Adjusted difference	
	Estimate (95% CI)	Estimate (95% CI)	p-value
Onlay	58.6 (58.5 to 58.8)	Reference	–
Retromuscular	58.7 (58.2 to 59.1)	0.1 (−0.5 to 0.5)	.943
Preperitoneal	59.5 (59.3 to 59.7)	0.8 (0.6 to 1.1)	< .001
Intraperitoneal	59.2 (58.9 to 59.4)	0.5 (0.2 to 0.8)	.001

Findings were consistent with the main analysis; however, intraperitoneal mesh was also associated with a higher AHQ sum score compared with onlay mesh, although the difference was small. AHQ, Abdominal Hernia-Q; CI, confidence interval.

Table S4

Abdominal Hernia-Q (AHQ) sum scores by mesh placement after exclusion of patients treated with Physiomesh™

	Adjusted mean	Adjusted difference	
	Estimate (95% CI)	Estimate (95% CI)	p-value
Onlay	57.7 (57.5 to 57.9)	Reference	–
Retromuscular	57.8 (57.3 to 58.3)	0.1 (−0.5 to 0.6)	.837
Preperitoneal	58.7 (58.4 to 58.9)	0.9 (0.6 to 1.2)	< .001
Intraperitoneal	58.0 (57.8 to 58.3)	0.3 (0.0 to 0.7)	.082

Findings were consistent with the main analysis. CI, confidence interval.

Table S5

Abdominal Hernia-Q (AHQ) sum scores by mesh placement adjusted for surgeon specialization level (specialist, non-specialist, supervised non-specialist)

	Adjusted mean	Adjusted difference	
	Estimate (95% CI)	Estimate (95% CI)	p-value
Onlay	57.6 (57.1 to 58.1)	Reference	–
Retromuscular	57.6 (56.7 to 58.6)	0.1 (−1.0 to 1.1)	.921
Preperitoneal	58.5 (57.8 to 59.2)	0.9 (0.1 to 1.7)	.028
Intraperitoneal	57.8 (57.1 to 58.4)	0.2 (−0.6 to 1.0)	.614

Information on surgeon ID and supervision was introduced into the Danish Ventral Hernia Database in 2017, resulting in missing data for earlier years. Surgeons were classified as specialists if they were registered in general or plastic surgery and had completed specialization at the time of repair. This subgroup analysis was based on complete cases without imputation for missing data (Table 1). Findings were consistent with the main analysis. CI, confidence interval.

Table S6

Abdominal Hernia-Q (AHQ) sum scores by mesh placement after exclusion of intraperitoneal mesh repairs without defect closure

	Adjusted mean	Adjusted difference	
	Estimate (95% CI)	Estimate (95% CI)	p-value
Onlay	57.7 (57.6 to 57.9)	Reference	–
Retromuscular	57.8 (57.3 to 58.3)	0.1 (−0.5 to 0.6)	.872
Preperitoneal	58.7 (58.5 to 58.9)	0.9 (0.7 to 1.2)	< .001
Intraperitoneal	58.1 (57.8 to 58.5)	0.4 (0.0 to 0.8)	.075

Findings were consistent with the main analysis. CI, confidence interval.

Table S7

Abdominal Hernia-Q (AHQ) sum scores by mesh placement using inverse probability weighting

	Adjusted mean	Adjusted difference	
	Estimate (95% CI)	Estimate (95% CI)	p-value
Onlay	57.7 (57.5 to 57.9)	Reference	–
Retromuscular	58.0 (57.5 to 58.6)	0.3 (−0.3 to 0.9)	.318
Preperitoneal	58.6 (58.4 to 58.9)	0.9 (0.6 to 1.2)	< .001
Intraperitoneal	57.8 (57.4 to 58.3)	0.1 (−0.4 to 0.5)	.808

Findings were consistent with the main analysis. CI, confidence interval.
